# Biological and Psychosocial Processes in the Development of Children’s Appetitive Traits: Insights from Developmental Theory and Research

**DOI:** 10.3390/nu10060692

**Published:** 2018-05-29

**Authors:** Catherine G. Russell, Alan Russell

**Affiliations:** 1 Deakin University, Faculty of Health, School of Exercise and Nutrition Sciences, Centre for Advanced Sensory Science, 221 Burwood Highway, Burwood, VIC 3125, Australia; 2 Flinders University, College of Education, Psychology and Social Work, Sturt Rd, Bedford Park, SA 5042, Australia; alan.russell@flinders.edu.au

**Keywords:** psychosocial processes, bidirectional processes, transactional processes, child, parenting, temperament, biological factors, appetitive traits, food neophobia, pediatric obesity

## Abstract

There has been increasing concern expressed about children’s food intakes and dietary patterns. These are closely linked to children’s appetitive traits (such as disinhibited eating and food fussiness/neophobia). Research has examined both biological and psychosocial correlates or predictors of these traits. There has been less focus on possible processes or mechanisms associated with children’s development of these traits and research that links biological and psychosocial factors. There is an absence of research that links biological and psychosocial factors. In the present article, we outline a model intended to facilitate theory and research on the development of appetitive traits. It is based on scholarship from developmental theory and research and incorporates biological factors such as genetic predispositions and temperament as well as psychosocial factors in terms of parent cognitions, feeding styles and feeding practices. Particular attention is directed to aspects such as emotional eating and feeding, self-regulation of energy intake, and non-shared family environments. We highlight the opportunity for longitudinal research that examines bidirectional, transactional and cascade processes and uses a developmental framework. The model provides a basis for connecting the biological foundations of appetitive traits to system-level analysis in the family. Knowledge generated through the application of the model should lead to more effective prevention and intervention initiatives.

## 1. Introduction

In contrast to dietary recommendations worldwide, large proportions of children consume lower than recommended amounts of, fruit, whole grains, low fat dairy and vegetables, together with higher than recommended amounts of refined grains, saturated fat, added sugars and sodium [1,2,3]. Children’s appetitive traits are one of a wide range of factors that influence children’s food intakes and dietary patterns. Appetitive traits develop in childhood and are precursors to dietary intakes which, in turn, contribute to outcomes such as food preferences [4,5] and weight [6,7,8]. There is now a rapidly emerging body of scholarship about the origins and development of appetitive traits in childhood which incorporates both biological and psychosocial processes.

In relation to biological factors, for example, it has been hypothesized that a genetic predisposition or temperamental susceptibility to higher weight gain or Body Mass Index (BMI) could be expressed via appetitive traits (described below) such as uncontrolled or disinhibited eating, lower satiety responsiveness (responsiveness to internal cues about whether they have had enough to eat) and higher food responsiveness (being attracted to food and eating) [9,10,11,12,13,14,15]. Food fussiness and food neophobia are also involved in children’s food intakes and dietary patterns, and here genetic factors have also been identified [16,17,18].

In addition to the identification of biological factors as contributors to children’s appetitive traits, there has been substantial attention to environmental contributions. The environmental emphasis has included an obesogenic environment (e.g., readily available and low cost high energy/low nutrient food supply, food advertising) [19] and psychosocial processes. The latter mostly pertain to parental factors such as their own food choices and their feeding practices as well as other elements of the family and home environment [20].

While research and theory about the development of children’s eating and weight has incorporated both biological and psychosocial processes relatively recently, this emphasis has been established in the field of developmental science, child development and socialization for decades. In the latter case, this has been formalized variously as a biopsychosocial approach [21,22,23,24] a developmental systems perspective [25,26,27,28] a transactional approach [29,30,31] or a bioecological model [32,33,34,35]. We use the term biopsychosocial throughout in reference to all four approaches that integrate biological and psychosocial influences on development.

In the present article, the purpose is to draw on the biopsychosocial approach in developmental theory and research to identify insights in relation to the development of children’s appetitive traits. In particular, we sought insights in terms of (a) the role of biological and psychosocial influences on children’s appetitive traits, (b) possible developmental processes or mechanisms arising from these two sources of influence, (c) relevant research designs, and (d) implications for measurement and data analysis. For illustrative purposes, we focus on two areas of child development and theory, namely adjustment problems in the form of conduct problems/externalizing behavior/behavior disorders, and self-regulation together with the associated construct of emotion regulation. The focus throughout is on conceptual analysis and development rather than an exhaustive examination of research findings.

The format of the present paper is a narrative review in which the emphasis is on possible mechanisms or processes associated with the development of appetitive traits arising from biological and psychosocial influences. The main age focus is on the period from infancy through early childhood and into middle childhood. As such, we are chiefly interested in the early origins and development of appetitive traits. Our approach was to examine the body of theory and research on the origins of children’s weight and eating through the lens of the more extensive scholarship in developmental science. Based on this examination we propose a process model of the development of appetitive traits. The model and associated research in developmental science is used to critically assess research on the development of appetitive traits, including research designs and data analysis. In both fields (children’s appetitive traits, and developmental science) we searched the literature from the 1950s and 1960s for core contributions in relation to research findings, theory development and model building. This involved (a) searching databases using key terms and associated concepts from the model, and (b) the major journals and research programs in children’s eating and weight and in developmental science. In developmental science we also sought review chapters in areas relevant to the model in another significant source of knowledge, namely “Handbooks” [21,33,36,37,38,39,40]. Most attention was directed to the age period from infancy into early middle childhood although pertinent research from other age periods was also drawn on.

The paper begins with a definition of appetitive traits, followed by a consideration of conceptual models that provide the foundation of developmental theory and research. Drawing on this work, we outline a biological and psychosocial model of the development of children’s eating and weight. The components and themes of this model shape the subsequent sections of the paper.

### 1.1. Children’s Appetitive Traits

Appetitive traits or eating behaviors have been described and measured via parent-report rating scales (e.g., a 5-point scale from “never” to “always”) in infancy and early childhood [41,42,43,44] and self-report ratings for older children e.g., [45]. Observational or experimental methods have also been used e.g., [46,47]. A number of eating behaviors and attitudes have been examined and there are several significant correlations among these traits e.g., [4,42]. For present purposes, we examine food neophobia and fussiness as a trait expressing food avoidance, and disinhibited eating as another group of traits expressing food approach. Under disinhibited eating we focus on the two most common forms, namely emotional eating and eating in the absence of hunger (EAH), and also links between these and satiety responsiveness or calorific self-regulation. Self-regulation of energy intake is an important appetitive trait that could play an important role in disinhibited eating.

Fussiness and food neophobia are two common forms of food avoidance (slowness in eating being another [42]. In general, fussiness pertains to reluctance to eat both new and familiar foods [17,48,49], while food neophobia relates to negative reactions to new or novel foods [50]. In this sense, food neophobia can be considered as a component of fussy eating. This overlap between the two is supported by Smith et al. [48] who concluded that there is a common genetic etiology to neophobia and fussiness. As could be expected, food neophobia and fussiness are consistently linked conceptually [51] and correlated empirically [18,48].

Disinhibited eating has been described as an overarching concept that incorporates both psychological and physiological components [52] and involves a lack of healthy restraint over energy intake [53]. Disinhibited eating was conceptualized by Shomaker et al. [53] as including EAH as the most frequently occurring disinhibited behavior followed by emotional eating, followed less frequently by loss of control eating and binge eating. In support of Barkeling et al. [54] who linked disinhibition and satiety responsiveness, Shomaker et al. argued that “these behaviors share in common a lack of reliance on physiological cues for hunger and fullness to determine initiation and/or termination of food intake” (p. 2192). They speculated that this common lack of awareness of appetitive cues could be combined with a greater susceptibility to external or emotional cues. A concomitant of this argument would be that disinhibition is associated with poorer self-regulation of energy intake.

### 1.2. Conceptual Models from the Fields of Developmental Science and the Development of Appetitive Traits

Advances in research and theory about child development as well as children’s eating and weight are reflected in the conceptual models used to summarize findings and/or provide a heuristic for further research. Conceptual models are central in the knowledge development process. Developmental science has a long history of conceptual model building, extending back more than 40 years, e.g., [27,28,34,55]. As we noted above, more recently these models or approaches have been variously described, but for present purposes we use “biopsychosocial”. A biopsychosocial approach incorporates three main sources of influence on developmental outcomes. The first are constitutional, genetic or biologically-based characteristics of the child, the second relate to the social/psychological and behavioral environment of the child (especially parenting, parent-child relationships and the family environment), and the third pertains to wider environmental, societal and cultural influences. The emphasis is then on systematically investigating how these factors and their complex interactions influence human health and development. Scholarship in developmental science has shown that incorporating biological foundations into the psychosocial factors and processes influencing development has been central to the advance of knowledge in recent decades.

Conceptual models associated with appetitive traits have dealt with parental feeding practices e.g., [56], learning processes with some child biologically-based influences [57], and influences from parents, the community and the macro-environment with acknowledgement of some innate preferences [58]. In Harrist et al.’s [59,60] model, several elements of interpersonal contexts and intrapersonal child mediators are linked to emotional and external eating, without a role for biological influences. A number of authors have argued that appetitive traits arise from genetic factors (such as predispositions) in interaction with the environment e.g., [19,61]. However, there do not appear to be comprehensive models with a focus on biopsychosocial processes over time. Other models of the development of children’s eating and weight e.g., [62,63,64] include the wider social, cultural and political environment. The wider environment clearly has an impact on elements pertaining to food, eating, and parenting. This third source of influence in the development of children’s appetitive traits is clearly important, but as a first step, attention in the present article is directed to biological and psychosocial factors (in particular parenting and parent-related processes) over time. The elements in the model are essentially content-free. For example, it does not indicate what kinds of beliefs or expectations the parent holds, or what foods they use in relation to emotional eating and feeding. This means that parental beliefs and practices could differ across cultures or social groups within the same process model. Other process models e.g., [59,65] appear to implicitly follow the same approach.

**Figure 1:**
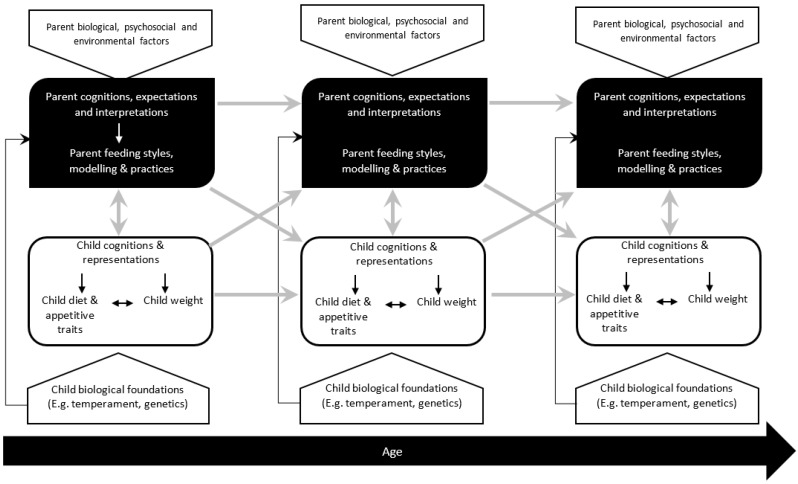
A model of biological and psychosocial processes in the early development of children’s eating and weight.

Drawing mainly on developmental theory and research, but together with theory and evidence about children’s eating and weight, we propose the biological and psychosocial process model of the early development of children’s eating and weight outlined in Figure 1. At the top of the model are parents’ characteristics and behaviors. At the bottom of the model are child characteristics and behaviors, with the two connected in a bidirectional fashion. Biological foundations (mainly in terms of genes and child temperament) are included and linked to child appetitive traits, food intake and weight, as well as to parents’ cognitions, feeding practices and feeding styles. The model assumes that appetitive traits are causally involved in weight gain and obesity. In addition, the model proposes a set of transactional influence processes over time. The main psychosocial influence processes in the model are (a) parents’ feeding styles and practices together with their associated cognitions, perceptions and attributions, and (b) parents as models through their own eating behaviors and food choices, and the family food environment. As we noted, although social, cultural and historical factors would be expected to have an impact on both parents and children, for present purposes the attention is directed to the biopsychosocial components of the model.

“Time 1, 2 and 3” in the model is intended to refer to measurement on at least three occasions in a prospective study, preferably over the period from infancy through early childhood and possibly beyond. This would ideally involve measurement of the same parent and child variables on each occasion, as in a cross-lagged panel research design. Kenny [66] argued that measurement on more than two occasions is important if the interest is in understanding influence processes over time. The model incorporates the potential for a more focused examination of processes that extend to moderators and mediators of influence processes. For example, child gender might moderate the relationship between parent perceptions of child temperament and emotional feeding. The general parenting relationship (e.g., level of warmth and acceptance) might moderate relationships between parent feeding and child appetitive traits. The link between child food neophobia at time 1 and directive feeding at time 2 could be mediated by parental perceptions and beliefs about the causes and consequences of food neophobia. The effect of child temperament on appetitive traits such as disinhibited eating could be mediated by self-regulation of energy intake. The effect of parent feeding practices on child weight gain is likely to be partly mediated by effects on child appetitive traits and food intakes. Finally, as noted in the model by Kremers et al. environmental factors could influence parent feeding styles and practices directly or indirectly through parental cognitions, expectations and interpretations. We note below that it is also important to take account of both conscious and implicit parental cognitions.

We now turn to a discussion and analysis of key features of the model, drawing on both developmental science and the literature on appetitive traits.

## 2. Biological Factors

### 2.1. A Biological Basis to Development

In developmental science, investigations of the biological basis of development e.g., [22,67,68] have mainly been directed at genetic contributions and child temperament. Both of these have received extensive coverage in research and model development in relation to conduct problems/externalizing behavior/behavior disorders e.g., [22,38,69,70,71,72,73,74,75,76,77,78]. This type of research is consistent with the consensus in developmental science about the benefit of a “biologically informed model of child functioning” [24] (p. 819). Research and theory in developmental science has also investigated genetic contributions to self-regulation and to environmental susceptibility [79]. In the case of self-regulation, special attention has been directed at the role of temperament, mainly through characteristics such as emotional reactivity, executive function, effortful control, emotion regulation, and inhibitory control [80,81,82,83,84,85,86,87]. Temperament pertains to individual differences in biologically-based and deeply-rooted predispositions towards emotional reactivity and self-regulation [37,88]. Emotional reactivity relates to the speed and extent of reactions involving both positive and negative emotions including fear and anger. Self-regulation covers attention, emotion and behavior.

With respect to emotion regulation, the focus has been on monitoring, evaluating and modifying emotional reactions, especially their intensity and duration [89]. The regulation deals mainly with self-related processes (such as self-relaxation or comfort eating), but also some external processes (e.g., parent cuddling child or using food to calm) [90,91]. It is important to note that self-regulation and/or emotion difficulties have been repeatedly linked to the development of conduct problems/externalizing behavior/behavior disorders [82,92]. The discussion below highlights the possible benefits of giving more attention to the role of temperamental characteristics such as emotion reactivity and self-regulation in the development of appetitive traits.

### 2.2. A Biological Basis to Appetitive Traits

With respect to biological foundations of appetitive traits, there is evidence about both of the two main possibilities, namely direct contributions from genes and via temperament.

#### 2.2.1. Direct Genetic Contributions to Appetitive Traits Involved in Disinhibited Eating

There is evidence of direct genetic contributions to satiety responsiveness and food responsiveness [93,94,95], enjoyment of food [96], disinhibition [95,97], eating in the absence of hunger [93,98]; emotional eating in adolescent and adult samples [95,99,100,101]; overeating amongst girls [102], loss of eating control [102], binge eating [95] and food intake self-regulation [93]. The direct genetic contributions to emotional eating could be more pronounced in adolescents and adults than young children [103,104]. Overall, therefore, there seems to be evidence for a substantial genetic underpinning of the traits and behaviors associated with disinhibited eating.

#### 2.2.2. Temperament and Children’s Appetitive Traits

Several dimensions of temperament appear to contribute directly to children’s appetitive traits. In particular, temperament has been associated with disinhibited eating (in particular EAH and emotional eating). Negative emotionality has been related to emotional eating [105]. Leung and colleagues [106] investigated direct contributions of three temperament dimensions to six obesogenic eating behaviors. They found differential associations of temperamental dimensions with disinhibited eating behaviors. Higher surgency was linked to overeating, desire to eat, pleasure from food and eating in the absence of hunger. Higher negative affectivity was linked to having tantrums over being denied food and being less likely to eat in the absence of hunger. No unique associations were found for effortful control.

Inhibitory control has also been found to be directly related to eating in the absence of hunger and weight outcomes in a number of studies by Birch and colleagues e.g., [107,108]. In a prospective study of girls Rollins et al. [108] showed that low inhibitory control was associated with a greater increase in EAH (and weight gain) from 5 to 7 years, but inhibitory control was not related to EAH at age 5. Consistent with Rollins et al., Riggs et al. [109] earlier had found that children lower in executive cognitive function such as inhibitory control and emotional control reported higher levels of snack food intake. 

Temperament has also been related to self-regulation, emotion regulation, and self-regulation of energy intake, which in turn are connected to disinhibited eating. Therefore, temperament could directly contribute to disinhibited eating or this effect could be mediated by effects on self-regulation of energy intake, for example, which has been shown to be a factor in disinhibited eating [56,59,60,93,110] and related to child BMI [111]. In support of this line of reasoning, Fisher et al. [112] proposed that difficulties in emotion regulation contribute to both emotional eating and uncontrolled eating. Given that emotional eating is related to responding to emotional states, especially so as to calm or control those states, it is reasonable to assume that temperamental qualities such as greater emotional reactivity or proneness to negative emotions (difficulties in emotion regulation) might be related to more emotional eating. This notion is consistent with Sim and Zeman’s findings [113]. They found that adolescent girls who experienced increased levels of negative affect, less emotional awareness and more difficulty coping with negative emotions reported more disordered eating, including emotional eating. These results highlight the importance of negative emotion, emotional reactivity or emotion regulation in disinhibited eating [112,114]. They add to results about the importance of self-regulation skills [85,115,116] in disinhibited eating and findings that disinhibited eating is connected with poor ability to self-regulate energy intake [56].

There is considerable overlap between high/low levels of disinhibited eating and high/low levels of self-regulation. In both cases, temperamental qualities such as levels of inhibitory control, executive cognitive function, and emotion regulation [111,116,117] are involved. Features such as lower executive cognitive function (including inhibitory control and emotional control) would be expected to contribute to lower self-regulation and could account for higher odds of being overweight/obese [115] and to more snack food consumption in children [109]. As Riggs et al. noted, executive cognitive dysfunction has been associated with a variety of dysregulated behavior such as conduct disorders, antisocial behavior and substance abuse. Their arguments provide support for the importance of further investigations of connections between executive cognitive dysfunction and disinhibited eating in order to better understand the processes associated with this connection.

It appears that temperament, then, can have both direct and indirect (through factors such as emotion regulation, self-regulation, and self-regulation of energy intake) effects on disinhibited eating. Temperament is clearly an important biological characteristic contributing to children’s appetitive traits related to disinhibited eating. 

#### 2.2.3. Temperament and Fussiness/Food Neophobia

Temperament has also been found to be related to children’s fussiness and food neophobia [118]. Powell et al. [119] found a greater emotional child temperament, which indicates a tendency to become aroused easily and intensely, was linked to higher food avoidance in 3- to 6-year-old children. Parallel results were reported by Haycraft and colleagues [120]. Additionally, Forestell and Mennella [121] found that infants higher on the temperament quality of approach consumed more beans, were less likely to show distaste and mothers perceived them as showing more enjoyment of the food. Recently, Holley et al. [122] also reported links between temperament (child sociability) and fussiness. Child sociability and food fussiness were related to vegetable consumption. Taken together, then, available results suggest that temperament is also an important biological factor contributing to fussiness and food neophobia.

### 2.3. Other Biological Factors Contributing to Development and the Development of Appetitive Traits

Beyond temperament and genetic factors, other biological factors could contribute to the development of appetitive traits. Similarities between parents and children in characteristics such as BMI or disinhibited eating might arise from common genetic factors, or from parent behavior and modeling or all of these. Any of these factors could explain a finding that mothers’ disinhibited eating was related to daughters’ EAH [123]. Hood et al. [124] argued that when parents were high on disinhibited eating this could foster weight gain in children, possibly through modeling or suppression of child’s self-regulation of intake. Other relevant biologically based characteristics include the universal predispositions to reject novel foods (neophobia) and prefer more familiar foods [125].

## 3. Biology Integrated with Psychosocial Processes

Biological susceptibility or predispositions (genetic and/or temperament) are implicated in child development outcomes such as behavior disorders and self-regulation, as well as children’s appetitive traits. Susceptibility or predispositions can be interpreted in terms of risk. Whether the biologically-based risk is translated into a developmental outcome such as an appetitive trait is dependent on environmental factors (such as type and availability of food), and psychosocial processes (especially connected to the family and parenting). In developmental theory and research, the psychosocial processes have been conceptualized in terms of bidirectional and transactional influence processes as well as developmental cascades.

The model in Figure 1 shows how child behavior and characteristics could be linked to psychosocial processes, both bidirectionally (from data on one occasion) and in a transactional process (over time). Transactional processes require cross-lagged measures (the same or closely parallel measures) on each occasion. Transactional processes extend bidirectionality to situations where each partner responds to emerging characteristics of the other in a feedback loop so that there is continual transformation in the behaviour, beliefs, goals etc. of both parent and child over time [126]. Research on children’s eating and weight could also be extended to examine possible developmental cascades if measures or outcomes outside of the domain of children’s eating and weight are included. For example, food neophobia could be measured at Time 1, parent-child conflict at Time 2 and peer-relationship difficulties at Time 3. With cascades, the notion is that characteristics of children (or parents) could have effects across other areas of development or functioning. In this case, food neophobia could have effects on parent-child conflict, and then on child peer relationships.

### 3.1. Bidirectional, Transactional and Cascade Influence Processes in Child Development Research

In this section we describe some illustrative examples of research investigating cascade, bidirectional, and transactional and processes from developmental science. This research is typically based on a developmental framework. Further, it is overwhelmingly multivariate and multi-method in design. Observation data are a key feature of much of this research.

#### 3.1.1. Cascade Processes

Davies et al. [127] examined cascades in relation to parental interpersonal dysphoria, child emotional insecurity and child psychological problems in five waves of data collection from age 7 to 15 years. For example, child emotional insecurity was related to increases in psychological problems and greater inter-parental dysphoria. Perry et al. [128] also investigated transactional and cascade processes, but this time associated with infant negative affectivity, child emotional reactivity and maternal intrusiveness over 3 waves of data collection from 5 months to 24 months. They found that infant negative affectivity (a temperamental characteristic) had effects on maternal intrusiveness that in turn had consequences for subsequent infant behavior and parenting. Improvements in early child executive function could yield a cascade of effects on parenting and contribute to beneficial social and behavioral outcomes for children, including school adjustment [86]. The recent literature contains many other examples of developmental cascades [129,130]. Elam et al. [129,131] raise possibilities of cascade effects on parenting and child development arising from child impulsivity or behavioral under-control that could extend into the domain of appetitive traits such as disinhibited eating. It should be recognized, however, that there are also likely to be effects of the early parent-child relationship (such as attachment security) on the development of self-regulation [132]. This suggests that cascade processes that extend into the domain of child eating and weight could be associated with early attachment security. 

#### 3.1.2. Bidirectional and Transactional Processes

The developmental literature contains numerous longitudinal studies investigating bidirectional and transactional influence processes [128,133,134]. In commenting on their results, Alemany et al. [134] referred to a downward spiral with child problem behaviors leading to increased parental negativity and in turn more child behavior problems. Perry et al. [128] found that infant negativity (experiencing and expressing emotions of frustration/anger, fear and sadness) was associated with later maternal intrusiveness and then with subsequent child negative affect. This was interpreted as the infant’s physiological functioning having an effect on their later behavior, which in turn had an impact on the parenting they received.

While cross-lagged panel designs have been used extensively in developmental science, other designs such as latent growth curve modelling and experimental manipulations such as cortisol reactivity measures have also been helpful in identifying possible developmental processes. These also generally include biologically-based child or parent measures together with psychosocial influences in a longitudinal design. For example, Barrios et al. [135] measured cortisol reactivity as a biological measure of child reactivity to stress at age 3, and Olson et al. [136] investigated trajectories of child externalizing problems from 3 to 10 in relation to executive control (a temperament variable), parental harsh discipline and maternal warmth.

#### 3.1.3. Developmental Framework

Developmental research typically takes account of and is cognizant of the course of and influences on development from infancy through childhood and beyond [68,137]. This is not possible with cross-sectional or short-term longitudinal research. Using a developmental framework means identifying and taking account of age-related changes in behaviour, competence and influences. This approach facilitates knowledge about the causal order of processes and outcomes, and emphasizes the influence of context on those processes and outcomes. It moves beyond the identification of risk factors or correlates to the examination of developmental processes. It can be extended to include a life-course perspective that takes account of effects on parents and families of life-stages and historical context and engenders an interdisciplinary approach to health, development and adjustment e.g., [138,139]

To summarize, in addition to showing the benefit of cross-lagged, biologically-informed, longitudinal research that incorporates psychosocial processes, if the mechanisms of development are to be elucidated, the developmental science literature points to areas that could be given more attention in relation to appetitive traits. Infant and child temperament variables such as emotional/negative reactivity and effortful control as well as self-regulation are highlighted as promising avenues. This is an extensive field of potential research and would include the investigation of direct and indirect links with appetitive traits as well as differences among temperament traits.

### 3.2. Bidirectional, Transactional and Cascade Influence Processes Associated with Appetitive Traits

There is now a large body of research about possible psychosocial influences on children’s appetitive traits. Most of this is cross-sectional rather than longitudinal, is not based on a cross-lagged design, does not draw on a developmental framework, or incorporate both biological foundations and psychosocial processes. A strength of the existing body of work on this topic, however, is that experimental designs have been used. We now examine the research on influences on children’s appetitive traits through the lens of research and theory in developmental science. First, we consider the research on disinhibited eating and then food neophobia/fussiness, with a focus on research pertaining to the variables and processes identified in Figure 1. This meant attention was directed to research relevant to questions about possible contributions of biological and psychosocial processes to the early development of appetitive traits.

Although some of the research on appetitive traits has involved experimental and observational measures, much of it is based on parental reports of their own and their child’s behavior. It is mostly not multi-variate and multi-method to the same extent as research in developmental science. The strong use of parental reports is despite mixed evidence about the validity of these reports e.g., [140,141,142,143,144] and arguments for the increased use of observational procedures e.g., [144,145,146]. Nevertheless, in the general field of children’s eating and weight, there is a growing body of observational research e.g., [115,143,147,148,149].

#### 3.2.1. Disinhibited Eating

From the evidence about biological foundations, it seems reasonable to assume that disinhibited eating arises as a predisposition that has an impact on parents and parenting behavior and that in turn is modified by experiences and is transactional. Evidence in relation to this assumption is suggestive but incomplete, as discussed below.

With respect to EAH, Birch and colleagues have published a series of papers on the effects of parental restriction and on EAH based on a longitudinal study of the health and development of young girls, commencing at age 5 with follow-ups at age 7, 9, 11, 13, and 15 [46,107]; Anzman and Birch, 2009; together with some independent studies [117]. Prompted by results that parental restriction increased girls’ EAH prospectively, these publications have generally claimed to support a parental influence position rather than a bidirectional position. Nevertheless, Birch et al. [46] reported a number of findings at age 5 indicating that parents were responding to child characteristics. In their discussion, they acknowledged that bidirectional processes are likely, but then went on to emphasize EAH as being promoted by higher levels of maternal restriction.

In later research [107,108,117], the group turned to possible biological foundations in terms of the two temperament dimensions of inhibitory control and approach. Interestingly, Rollins et al. [109] acknowledged that children with low inhibitory control might elicit more controlled/restrictive feeding practices, but instead argued for a position that children low on inhibitory control could be more susceptible to the negative effects of restriction. In their series of studies Birch, Rollins and colleagues did not appear to examine the possibility of a transactional process where feeding practices are evoked by child characteristics which in turn increase EAH and then more restriction. With a main focus on how restriction predicted EAH, these publications did not indicate whether increased levels of restriction were predicted by child EAH. The fact that the baseline for the research was at age 5 limited the possibilities to examine the early origins and processes associated with EAH and to use a broad developmental framework.

The early origins of EAH could be associated with a combination of genetic predispositions, temperament and parent behavior. With respect to the latter, parents’ high on disinhibited eating could model EAH or suppress the child’s self-regulation of energy intake [124]. Mother modeling of dietary disinhibition could be more closely related to EAH in girls than in boys [47]. The role of parent as models would extend to the food environment of the home as well as their expression of conscious and unconscious beliefs and expectations about food and diet (the modeling scales developed by Palfreyman et al. [150] show ways in which parents could provide models). The food environment includes the availability of energy-dense foods, the types and availability of snack foods, and portion sizes e.g., [151,152]. Parent and child disinhibited eating clearly is related to the family and wider social and psychological context. Nevertheless, there has been a primary focus on the role of parent eating behaviors and feeding practices in the development of disinhibited eating.

Whereas much research on EAH has commenced with children at approximately age 5, with follow-ups, research on emotional eating has covered a wider age range, including infants, young children and older ages. A number of psychosocial processes have been associated with emotional eating, but particular attention has been directed to emotional feeding [153,154] and use of food as a reward [155,156]. However, the research on this topic has mostly not been longitudinal, has not examined bidirectional and transactional processes and has not incorporated both biological and psychosocial factors. It has generally been conducted within a framework of parental influences. Nevertheless, it has helped to clarify likely psychosocial processes that could be implicated in the development of emotional eating.

For example, in a cross-sectional study with 5-year-old twins and their parents, Herle et al. [104] reported that the main contribution to emotional eating was from shared family environments. Features of the environment were not measured, but it was argued that emotional feeding was a probable factor and that emotional eating was learned, not inherited. Herle et al. [104] argued that it is likely that parents who emotionally overeat will create an environment which nurtures this behavior in their children, partly through emotional feeding. Emotional feeding could teach children maladaptive strategies in response to stress [157] and to use food to regulate emotions [114,158]. The effects of parents on children’s emotional eating has also been explored in relation to the use of food rewards. In a two-year follow-up of 6-year olds, Steinbekk et al. [156] showed that greater parental use of food as a reward predicted more emotional eating 2 years later.

The link between parents’ and children’s emotional eating could be somewhat nuanced, though. Tan and Holub [154] found that the association between parents’ and children’s emotional eating was mediated by parents’ use of emotion regulation feeding practices. However, this association only occurred for children who were low on self-regulation in eating. Put another way, children who were better able to self-regulate their eating seemed to be less impacted by parent’s emotion regulation feeding practices. Another complexity is that by adolescence, a genetic predisposition might interact with environmental conditions in leading to emotional eating. For example, depressive feelings might be associated with a greater increase in emotional eating only in connection with a genetic vulnerability [159], and parental control might be associated with a higher increase in emotional eating only in connection with the DRD2 genotype [101]. 

If emotional feeding is a factor in the development of emotional eating, it is important to know more about factors contributing to emotional feeding and its possible origins in early childhood. There is evidence that emotional feeding begins in infancy and is probably linked to infant temperament. McMeekin et al. [160] found that mothers of 2–7 month-old infants were more likely to use food to calm the infant if he/she had a difficult temperament. They derived an overall easy-difficult temperament measure from the three scales of approach, cooperation and irritability. Difficult temperament infants react negatively to new situations, unfamiliar people and novel activities. It is apparent, therefore, that difficult temperament early in infancy could be a factor in the early origins of using food for emotion regulation as well as parenting style and parent feeding style. Longitudinal research with a cross-lagged design would assist in clarifying influence processes beyond infancy. That is, to clarify whether and how use of food for emotion regulation is linked to emotional eating in children.

In a related way, it is apparent that children’s difficulties in emotion regulation contribute to their emotional eating [112,113], and these difficulties presumably contribute to emotional feeding to help regulate child emotions. This suggests some “child effects”, where parents respond to child behavior or characteristics. For example, Vollrath et al. [161] found that at 18 months, mothers were more likely to offer sweet food and drinks to children with either internalizing or externalizing difficulties. Powell et al. [119] found that a child’s emotional temperament was associated with more maternal feeding control, greater use of food for behavior regulation and lower encouragement of a balanced and varied food intake. In this way, young children are exposed to the use of food to regulate emotions and behavior. In a cross-lagged biologically based study Steinsbekk et al. [162] measured negative affectivity at age 4 using the Children’s Behavior Questionnaire (CBQ) [163]. The results showed a cascading developmental sequence in which negative affectivity contributed to both emotion eating and feeding at age 6, followed by a bidirectional sequence with emotional eating contributing to emotional feeding, and emotional feeding contributing to emotional eating at each age point. Their results are consistent with the main theme in the present article, namely that a biologically-based child characteristic (in this case negative affectivity) can provide the foundation for bidirectional and transactional developmental processes involving emotional eating and feeding.

As presented in Figure 1, parental cognitions (such as interpretations of child behavior, beliefs about children and parenting etc.) are important in how parents respond to and interact with children about food and eating. This is especially apposite in relation to child emotions and parents’ use of food to regulate child emotions. Meyer et al. [164] investigated parental beliefs and values about emotions in a sample with 4-year-old children. These were beliefs about the importance of attending to and accepting emotions and beliefs about managing negative moods and maintaining positive emotions. These beliefs were associated both with parental socialization strategies and children’s use of emotion regulation strategies. Morey and Gentzler [165] examined parents’ perceptions of and responses to children’s emotions. Johnston et al. [166] showed that there is a need to examine the role of both conscious and implicit parent cognitions in evocative process associated with child behaviors. Conscious cognitions pertain to reportable explicit attributes and attitudes, whereas implicit cognitions are more automatic and uncontrolled. Some parental cognitions about child emotions and how to respond to them could be automatic and uncontrolled or unconscious.

Drawing on some of the findings about parental cognitions, it is apparent that there is scope for more attention to the role of cognitions such as beliefs, expectations and interpretations of child behavior and the parental role in research on parents’ use of food in regulating children’s emotions. 

#### 3.2.2. Self-Regulation of Energy Intakes

Lower ability to self-regulate energy intake is clearly a factor in disinhibited eating. As a consequence, considerable research has been directed to the development of self-regulation of energy intake. Biologically-based child characteristics have been examined. They include temperamental features such as delay of gratification, self-regulation, inhibitory control and executive function. These are important characteristics as they have been linked to BMI and weight gain [167,168,169].

Effort has also been directed to the role of child characteristics and parenting in the development of self-regulation of energy intake. This has included parent styles and practices such as responsive feeding in infancy [170], authoritative parenting and parent responses to child negative emotions [171], use of food as a reward [151,172], controlling feeding practices [119], and pressure to eat [151,173]. Controlling or restrictive feeding practices have been found to reduce children’s ability to self-regulate energy intake [116,174,175]. When parents believe that their children can self-regulate, they use less restrictive feeding practices [176].

Conclusions and evidence about the importance of self-regulation in child development (including in relation to eating and weight), strengthens the argument for greater attention to the developmental course, origins and influences on self-regulation of energy intake in a way that parallels the extensive research on self-regulation in the child development area e.g., [83,84,177]. What would be beneficial is not just research on how self-regulation influences eating and weight or what contributes to self-regulation of energy intake, but attention to the developmental course of self-regulation of energy intake over the early years. Zeytinoglu et al. [178] noted that “by 5 years of age, most children demonstrate an increasing capacity for regulating their own arousal, attention, emotional responses, cognitive processes, and goal-oriented behaviors” (p. 170). Advances in child executive functioning in early childhood could initiate a cascade of effects on parenting and contribute to beneficial effects for children [86]. It would be valuable to examine these capacities in relation to children’s food, eating and energy intake as well as their impact on parents, including feeding practices. It is important to recognize that developmental change is associated with adjustments by parents to changing child behavior [179].

#### 3.2.3. Food Neophobia/Fussiness

There has been considerable research on psychosocial processes related to children’s food neophobia/fussiness. Much of this research is cross-sectional e.g., [16,180,181,182], but there are examples of research that has incorporated both biological foundations and psychosocial processes in a longitudinal design. For instance, Moding and Stifter [183] assessed child temperament (approach/withdrawal plus positive and negative affect) via observations at 18 months and 4.5 years together with parent reports at age 4.5 years when food neophobia was observed and reported by mothers. Mothers also reported their feeding practices. Temperament (e.g., negative affectivity) was related to food neophobia at 4.5 years. Temperament at 18 months (e.g., low approach, high negative affect to novelty, low positive affect to novelty) was also associated with neophobia at 4.5 years. These results add to the evidence reported above (Section 2.2.2) that temperament traits are linked with food neophobia. The one feeding practice related to neophobia was pressure to eat. However, the associations between pressure to eat and neophobia differed according to child temperament. They argued that pressure to eat could lower the risk of neophobia in children with low negative affect to novelty (a temperament characteristic), but increase the risk of neophobia in children with high negative affect to novelty. So, pressure to eat could lessen neophobia in some children and increase the risk in other children. This idea is consistent with the notion of moving away from a “one-size fits all approach” [184]. The Moding and Stifter [183] results supports arguments for longitudinal cross-lagged research that includes temperament, parenting and measures of neophobia.

Jansen et al.’s longitudinal results [185] suggest possible transactional process involving pressure and fussy eating. They found that fussy eating at 1 1/2 and 3 was related to pressure to eat at age 4. Pressure to eat at age 4 predicted increased fussiness at age 6. The relationship between fussy eating at age 3 to parenting at 4 was stronger than pressure to eat at age 4 to fussy eating at age 6. This set of findings is consistent with a transactional process. Further research could examine pressure to eat prior to age 4 and include biological foundations in terms of temperament before age 1 ½. This research might also include offering new foods as a factor potentially related to lower neophobia, as well as evidence about the parent’s own food neophobia [180,186].

Clarification is needed about whether pressure to eat and other feeding practices or feeding styles have their origins in very early temperament characteristics such as fearfulness and negative affect associated with novelty. Cassells et al. [186] thought that the origins of pressure to eat could be found in early concerns about infant under eating and being underweight (they measured these at 4 months). Others have identified pressure to eat related to concerns about child being or becoming under-weight as well as reports of higher levels of child food fussiness [187]. In common with Cassells et al., these studies typically have not included or taken account of infant temperament. A possible hypothesis is that early temperamental approach-withdrawal (fearfulness) tendencies have an impact on the developing concerns of parents about child eating and weight that contribute to pressure to eat that in turn contributes to the emergence of food neophobia (which might be an expression of fearfulness in the food area or negative reactions to food [118]). Food neophobia, then, could increase pressure to eat related foods, which then increases food neophobia in a transactional process over time. In proposing a set of processes such as these, it should be kept in mind that the early pressure to eat could partly arise from cultural factors and/or parent characteristics such as eating disorders.

Faith et al. [16] found that the main environmental effects on food neophobia could arise from the nonshared family environment. This means that food neophobia could be linked to parents’ differential treatment of siblings. Differential treatment is likely to extend to other appetitive traits. For example, Tripicchio et al. [188] found that mothers differed in the feeding practice of restricting their child’s access to food even between same-gender twin pairs. Differential treatment is a phenomenon that has a long history of research in child development [189] together with the concomitant concept of nonshared aspects of family environments [190]. In developmental sciences this theme was early articulated in the now seminal article from Plomin and Daniels [191] entitled “Why are children in the same family so different from one another?” In a follow-up article Plomin et al. [192] suggested that the nonshared environment arose partly from differential treatment from parents, but also from children’s differential reactions to aspects of the shared environment. For example, siblings could respond differently to the same pressure to eat practices of parents.

Collectively, the developmental science research and findings by Faith et al. [16] suggests that to some degree there is a specific food environment for each child in the family. Faith et al. argue that certain food exposure strategies could be effective in increasing food acceptance for one sibling with a different strategy increasing acceptance in other children. This idea raises the possibility that bidirectional and transactional influence processes within families could to some extent be child-specific. An associated argument was presented by Faith et al. [174] who suggested that restrictive food practices could have their greatest effect on children predisposed to overweight. Again, the idea is that influence processes might differ at least according to certain child characteristics. The analyses of genetic and environmental contributions have tended to show that the environmental effects arise more from the nonshared environment than from the shared environment [193]. This means that the environment for each child within the family is somewhat unique.

#### 3.2.4. Developmental Cascades in Children’s Eating

At present, developmental cascades do not appear to have been examined in the literature on children’s appetitive traits. There seem to be two possibilities. One relates to cascades into the area of eating and weight. For example, how infant temperament or family relationships might have effects across domains, including eating and weight. A second direction pertains to how children’s appetitive traits such as food neophobia could have effects and consequences across other domains of development.

In summary, both biological and psychosocial factors clearly contribute to the level and development of food neophobia/fussiness. The extensive cross-sectional research on psychosocial factors has a number of suggestive findings. There is only a small body of research that is longitudinal and that considers bidirectional and transactional processes although cascade effects stemming from or contributing to food neophobia/fussiness do not appear to have been considered.

### 3.3. Developmental Framework

Research on children’s appetitive traits have spanned developmental periods from infancy to early childhood and beyond. However, most of this research has not been cognizant of a life-course perspective and has not been based on a developmental framework in relation to changes and processes across the ages from infancy and childhood.

Nevertheless, there has been interest in developmental changes in appetitive traits. For example, food neophobia has been found to decrease more rapidly with infants than with children [10] and there is evidence that rejection of novel foods in infancy predicts food neophobia in early childhood [183]. Hittner and Faith [194] examined subgroups of emergent eating patterns in children from ages 1 to 3 years. Efforts have also been made to track appetitive traits between 4 and 10 years, showing small-to-moderate changes across these ages [44]. For example, Johnson [57] cited Grimm et al. [195] in claiming that vegetable acceptance peaked in infancy, declined after 1 year, and continued to fall throughout the preschool years. Johnson went on to propose a model of effects on children’s vegetable preferences and consumption drawing on influences and changes across the early years. This is an important illustration of how a developmental framework can assist in identifying processes in the development of appetitive traits and food neophobia.

## 4. Insights, Implications and Conclusions

Our purpose in this review was identify insights from development science for research and theory about the development of children’s appetitive traits. The review first highlighted the significance of what has been described as a biopsychosocial approach in developmental science. We used this approach to construct a process model of the development of appetitive traits in infancy and early childhood. This model integrated biological and psychosocial influences. The model and associated research in developmental science showed the importance of bidirectional, transactional, and cascade influence processes. We examined the research on the development of appetitive traits against the biopsychosocial approach and associated influence processes. It was apparent that although biological and psychosocial influences have been identified in relation to the development of children’s appetitive traits, these two types of influence have mostly not been integrated in a way consistent with a biopsychosocial approach. We noted some suggestions of bidirectional processes but there was more of an emphasis on a “parental influence model”. Some data hinted at a transactional process, but mostly the research has not focused on the possibility of transactional or cascade processes. The model provides a rich source of testable hypotheses about processes in the development of appetitive traits in childhood.

### 4.1. Methodological Approaches

There seemed to be a contrast between research in developmental science and the development of children’s appetitive traits in relation to research designs. In the former case, multi-variate, multi-method longitudinal research is prominent. This has enabled data analysis strategies that can focus on possible mechanism and processes over time. In contrast, much of the research on the development of children’s appetitive traits has been cross-sectional, or involves a relatively short-term longitudinal design, and has used questionnaire reports of parent and child behavior. An implication is that further advances in knowledge about the development of appetitive traits could be expected if that research was able to move more towards the kinds of design, measurement and data analysis approaches that are common in developmental science.

There are a number of other insights and implications that arise from the present review.

### 4.2. Self-Regulation of Energy Intake

In our discussion of disinhibited eating, self-regulation of energy intake was a prominent theme. We argued for greater attention to its role and development in relation to disinhibited eating. In 1996 Birch and Fisher [196] encouraged parents to focus less on their immediate concerns about food composition and quantity of food and instead pay attention to the long-term goal of developing healthy self-regulation of energy intake. It might have been expected that the next decades would have shown a strong orientation to research on the development of self-regulation of energy intake. It is probably fair to say that this has not happened. In 2012 Frankel et al. [116] took up the theme and argued that there were helpful links between the child development literature on emotion regulation and self-regulation of energy intake. Again, however, their suggestions did not seem to be associated with an upsurge in research on the development of self-regulation of energy intake in early childhood. More attention to self-regulation of energy intake could be important for advances in knowledge about EAH and emotional eating. Saltzman et al. [197] have recently made a similar argument, although in their formulation the likely biological bases to self-regulation were not incorporated.

In the developmental research, there have been methodological advances that could assist in research on self-regulation of energy intake. Parent reports have been used to assess effortful control in toddlers based on Rothbart’s Child Behavior Questionnaire [198]. Tasks have been developed to assess executive function in early childhood [199], effortful control in toddlers [136,200], self-regulation in early childhood [201,202], including the Dimension Change Card Sort task [86]. These tasks and procedures could be used in research on the development of self-regulation of energy intake. In addition, observational procedures have been used to measure children’s emotion regulation from infancy to age 5 [203] that could be used to examine processes associated with the development of emotional eating.

Research and theory on emotion regulation and emotion socialization has been a prominent theme in the developmental literature for decades [204]. Much of this research has involved observational methods of mothers’ reactions to children’s emotions e.g., [205]. There is an opportunity for research on parent emotional feeding and child emotional eating to draw on methods, research designs and theoretical perspectives in the developmental literature. 

### 4.3. Differential Parental Treatment

The developmental science literature has had a concentration on differential parental treatment and nonshared environments in the family for several decades, a possibility that has been referred to at times in the literature on children’s eating and weight e.g., [16,188,206,207]. This idea should be combined with the likelihood that child characteristics (such as temperament) serve to evoke reactions and feeding strategies from parents, and that different children in the family might react differently to the same parental feeding practices, to suggest that the food-related environment within families could be somewhat unique for each child. This possibility clearly has implications both for research design and theory construction.

### 4.4. The Design of Intervention Strategies

The possibility of differential parental treatment has implications for the design of intervention strategies, where we noted arguments against the approach of “one-size fits all” [184]. The components of the model in Figure 1 and the associated influence process also appear to have implications for intervention strategies. For example, it suggests that the knowledge-base for interventions should come from influence processes and mechanism in addition to risks factors. Furthermore, interventions could profitably cover the different features of the model individually (such as child temperament or parental cognitions) and how they impact the targeted outcomes (such as obesity, disinhibited eating or food neophobia).
